# Association of the Length of Service in the 24/48 Shift of Firefighters of the State Fire Service in Wroclaw on Selected Serum Biochemical Parameters of Nutritional Status

**DOI:** 10.3390/nu16152467

**Published:** 2024-07-29

**Authors:** Karolina Dobrowolska-Zrałka, Łucja Janek, Lilla Pawlik-Sobecka, Jacek Smereka, Bożena Regulska-Ilow

**Affiliations:** 1 Department of Dietetics and Bromatology, Wroclaw Medical University, ul. Borowska 211, 50-556 Wroclaw, Poland; bozena.regulska-ilow@umw.edu.pl; 2 Statistical Analysis Center, Wroclaw Medical University, ul. K. Marcinkowskiego 2-6, 50-368 Wroclaw, Poland; lucja.janek@umw.edu.pl; 3 Division of Basic Medical Sciences, Wroclaw Medical University, ul. Borowska 211, 50-556 Wroclaw, Poland; lilla.pawlik-sobecka@umw.edu.pl; 4 Department of Emergency Medical Service, Wroclaw Medical University, ul. Parkowa 34, 51-616 Wroclaw, Poland; jacek.smereka@umw.edu.pl

**Keywords:** firefighters, shift work, lipid profile, lipid ratio, NRF9.3 index

## Abstract

The aim of the study was to evaluate the association of the quality of diet as calculated by the Nutrient Rich Food index (NRF9.3), and length of service (LS) (≤10 years vs. >10 years) with selected serum biochemical parameters, the proportions of different lipid profile fractions and advanced glycation endproduct (AGE) values of 108 firefighters from the State Fire Service in Wroclaw. The LS officers > 10 years had significantly higher total cholesterol (211.50 (184.00–254.00) vs. 184.00 (166.00–194.00)), LDL (123.75 (108.20–167.90) vs. 105.18 (90.24–119.00)) non-HDL (151.70 (132.00–196.70) vs. 122.00 (106.00–140.00)), triglycerides (118.50 (96.00–158.00) vs. 78.00 (67.00–103.00)) and lower HDL concentrations (51.30 (45.60–56.70) vs. 58.00 (51.70–66.10)) compared to firefighters in the LS ≤ 10 years group. Significant differences between the seniority groups were also noted for all lipid profile ratios. Regardless of the officers’ seniority, systolic blood pressure was observed at the highest normal level of 134.4 ± 14.4 in the LS ≤ 10 years group and 139.5 ± 14.3 in the LS > 10 years group. Advanced glycation endproduct values were significantly dependent on diet quality, as expressed by the NRF9.3 index and on the TG/HDL ratio, but not on seniority. Diet quality, as expressed by the NRF9.3 index, had a significant association with GLU and FI levels, and components of the lipid profile between seniority groups. As NRF9.3 increased, TG/HDL, LDL/HDL, TC/HDL, and non-HDL/HDL ratios decreased. AGEs were significantly affected by NRF9.3 and significantly associated with TG/HDL. Firefighters’ diets, as assessed by the NRF9.3 index, had a significant association with predictors of insulin resistance, diabetes, and cardiometabolic predictors between seniority groups. The nutritional education of firefighters (and other professional groups working irregularly), especially those with longer tenure (e.g., >10 years), is necessary to prevent the development of, e.g., CVD, MetS, and T2DM, which contribute towards a reduced ability to perform professional duties.

## 1. Introduction

The literature encompasses an increasing number of reports indicating the impact of shift work on the health of workers in various professions, such as medical, transportation, industrial, and uniformed services [1]. According to an assessment of the impact of shift work on health by the International Agency for Research on Cancer (IARC), seniority and frequency of night work are the most crucial factors affecting metabolic rates. It is associated with disrupting the day–night cycle, the light–dark cycle, limited opportunities for regular and rational nutrition [2], interference with biological functions, and psychosocial issues [3].

Firefighters, with the additional risks associated with their duties such as above-average physical exertion, physical and psychological trauma [4,5,6], toxic fumes [7], and stress [8,9], which are risk factors for cardiometabolic diseases, are an under-researched group in this aspect. As an example, it was observed that the leading cause of death for American firefighters on duty was sudden cardiac arrest and psychological stress, as effects of post-traumatic stress syndrome [10].

Based on a systematic review, Barros B. et al. [11] noted an increased incidence of malignancies, cardiovascular disease (CVD), and respiratory disease in firefighters compared to men in the general population. Elevated biomarker levels of early inflammation were observed in firefighters compared to those of a control group; these are considered a primary cause of metabolic disorders such as insulin resistance [12].

According to the IARC, the incidence and amount of nighttime work are the principal factors to be considered in the biochemical analysis of the human body when assessing the impact of shift work on the health of workers. It is then possible to estimate the effects (more or less severe) and the impact of various shift systems on human health, through interference with biological functions and psychosocial issues [3].

Scientific studies of firefighters that evaluate the concentration of individual biochemical parameters are few and mainly concern the presence of toxic substances from fire smoke [7,13], or the analysis of lipid profiles, in the context of the diagnosis of metabolic syndrome (MetS) and/or cardiometabolic diseases [14,15,16]. To the best of our current knowledge, there are only a few studies in the world on nutrition assessments of firefighters; the most similar to the current study is that carried out by Romanidou M. et al. [17], which aimed to evaluate the association between adherence to a modified Mediterranean diet and the incidence of MetS based on cross-sectional associations with the anthropometric indices, blood pressure, and biochemical parameters of active American firefighters. However, in the cited study, the analysis of the diets was conducted using the Food Frequency Questionnaire (FFQ) and the modified Mediterranean Diet Score (mMDS); the biochemical tests included only lipid profiles and fasting glucose levels. 

Torre S. et al. [18], in their nutritional intervention, noted that firefighters had an unbalanced diet that was dominated by low-quality products; the diets did not meet the fiber and micronutrient requirements of national guidelines. For firefighters, the main barriers to adhering to proper nutrition included a lack of time.

The main objective of our study was to evaluate the association of firefighters’ diet quality, assessed using the Nutrient Rich Food 9.3 (NRF9.3) index, and length of service (LS) (≤10 years vs. >10 years), with selected serum biochemical parameters and advanced glycation endproduct (AEG) values.

## 2. Material and Methods

### 2.1. Participants of the Study

The invitation to participate in the study was received by all men employed in eight Fire and Rescue Units of the State Fire Service around the city of Wroclaw, Poland, constituting a group of 383 men. The men worked in a 24/48 shift system (24 h on duty, 48 h off); additionally, during the month they had so-called “free duty”, meaning a day off when other colleagues from the shift were in the unit.

Special information brochures with an invitation to participate in the study were prepared to provide information about the study. They were delivered personally to each unit of the State Fire Service in the Wroclaw area, with a simultaneous brief informational meeting with the Commander and firefighters on duty at the respective unit. At the meeting, the purpose of the survey, the benefits of participating in it, and its various stages were discussed. During the meeting, questions asked by firefighters were additionally answered and a date was set for the firefighters to come to the unit to take the measurements.

The inclusion criteria were men employed in the State Fire Service, in Rescue and Fire Fighting Units in the City of Wroclaw and who performed work in the 24/48 shift system. No exclusion criteria were applied in the study (including the therapies used by the study participants), as firefighters undergo regular medical examinations and must not have diseases that affect their fitness and aerobic capacity (including CVD). Otherwise, they are redirected to daytime work in offices. 

A total of 133 men, representing approximately 34.7% of all firefighters employed in the units, were willing to participate in the study, and 130 of them prepared a 3-day food diary and took part in the study of final glycation products using an AGE reader device.

Biological material was collected from 108 study participants (81.2% of enrolled men and 30% of all firefighters employed in the Rescue and Fire Fighting Units of the State Fire Service in Wroclaw). The study participants were divided into two groups, relative to LS. The group with short LS included participants performing their professional duties ≤ 10 years, while those with long LS > 10 years comprised the other group.

### 2.2. Method of Data Collection and Research Methods Used

Approval to conduct a scientific study was granted by the Bioethics Committee at the Wroclaw Medical University, (No. KB–760/2020, No. KB-767/2021 and No. KB–926/2021). The research was supported by Wroclaw Medical University Research Grants (SUB.E.110.21.007 and SUB.E080.19.013).

The study was conducted between November 2021 and April 2022. Dietary interviews and anthropometric measurements were carried out at the respective Fire and Rescue Unit for all 3 shifts. On the day of the study, each volunteer signed a consent form to voluntarily participate in the study. All 360 firefighters in the city received an invitation to participate in the study. Of these, 130 (30% of all men) expressed their willingness to participate in the survey. We therefore consider the results of this survey to be representative of the entire group of firefighters in the city.

The men were subjected to body composition analysis (body weight, muscle tissue and body fat, BMI, and WHR) using the Accuniq BC310 portable body composition analyzer (Selvas Healthcare, Sinseong-ro, Yuseong-gu, Daejeon, 34109, Republic of Korea), measurements of height with the TANITA HR-001 mobile height meter (TANITA, Tokyo, Japan), waist circumference using the BMI GIMA measuring tape, and a blood pressure test with the Omron Healthcare Co., M6 Comfort, HEM-7360-E (Kyoto, Japan). Each participant in the study was also analyzed for advanced glycation endproducts using a non-invasive AGE reader device (Diagnoptics Technologies B.V. Aarhusweg 4-9, 9723 JJ, Groningen, The Netherlands). Before the measurements were taken, the devices were prepared according to the manufacturers’ recommendations.

The detailed method of collecting and analyzing the data obtained in the dietary interview and how the NRF9.3 index was calculated is described in the published article [19].

### 2.3. Biochemical Analysis of Blood Serum

All study participants underwent medical qualification. Men with no contraindications to laboratory tests were referred for serum tests, which included glucose, insulin, TSH hormone, and lipid profiles. Men were required to report to the blood collection facility with an empty stomach.

Venous blood samples were collected from each participant at the blood collection facility by qualified medical personnel. K3EDTA anticoagulant tubes were used to obtain whole blood.

To obtain plasma for glucose determination, venous blood was collected into a tube with an anticoagulant [sodium fluoride]. After centrifugation of the test sample, glucose in the fluoride plasma was determined by spectrophotometry using a cobas^®^ Pro c503 analyzer (Roche Diagnostics at F. Hoffmann-La Roche Ltd., Basel, Switzerland).

Serum was obtained by collecting venous blood into a tube with a clotting activator. After clotting and centrifugation, a portion of the serum was used for the lipid profile.

The remaining serum was separated from the clot and dissociated into Eppendorf Tubes^®^ 3810X (Eppendorf, Hamburg, Germany) then frozen at minus 20 °C. Once adequate samples were collected, ELISA tests were performed. Insulin concentrations, as well as TSH, were determined using Calbiotech. ELISA-based tests were performed in accordance with good laboratory practices.

## 3. Statistical Analysis 

The assumption of normality of distribution was checked (for *n* ≤ 50 using the Shapiro–Wilk test and for *n* > 50 using the Kolomogrov–Smirnov test with a Lilliefors correction for the Student’s *t*-test). When the assumption of normality of the distribution was not met, the median and quartiles were presented and the Mann–Whitney U test (hereafter referred to as the M–W test) was applied. When the assumption of normality of distribution was met and the assumption of homogeneity of variance was not met, the Welch-corrected Student’s *t*-test was used. When the assumptions were met, the mean and standard deviation were presented and the Student’s *t*-test for independent pairs.

The association of a pair of variables (a continuous variable and a nominal variable) on single continuous variables was tested using a non-parametric analysis of covariance (hereafter referred to as ANCOVA). The assumption of normality of the distribution of the explanatory variable for the parametric ANCOVA was tested. The assumption was not met. For a more insightful interpretation, the data were not transformed and other assumptions were checked: the assumption of normality of the distribution of the explanatory variable; the assumption of homogeneity of the variance of the explanatory and explanatory variables; the assumption of the equality of regression slopes and a non-parametric ANCOVA using R (version 4.3.1) in the WRS2 package function “fitanc ← ancova(‘HSA’~LS (0–1) + ‘Age (0–1)’ + ‘BMI (0–2)’ + ‘WHR (0–1)’ + ‘NRF9.3’, data = data) fitanc”. Ref. [20] from the WRS2 package with R Studio [20].

The influence of a pair of continuous variables and a nominal variable on the ordination variable was examined using ordinal multinomial logistic regression. Associations were checked as follows: the assumption of no outliers and outliers was removed; the assumption of no multicollinearity of predictors (using Spearman correlation); and the assumption of linearity between the predictors and the logit of the predictors (using the Box–Tidwell test) for logistic regression. All associations were summed, parameters were assessed, odds ratio (OR) and goodness of fit of the model were calculated (using the Akaike information criterion—AIC) (R).

Qualitative variables were tested using Pearson’s chi-squared test of independence. The assumption of expected abundance (*n* < 5 in ≤20% of cells) for the chi-squared test was met (Statistica).

Variable changes in the use of Spearman correlations were examined. The assumption of normality of distribution and the assumption of homogeneity of variance for Pearson’s correlation were not met.

Assumption checking and intermediate calculations are presented in the Supplementary Materials.

Due to comparisons, the Dunn–Sidak correction for the M–W test (excluding characteristics) and α = 0.05 were imposed.

Statistical analysis was performed using Statistica PL 13.3 from StatSoft (StatSoft Inc., Tulsa, OK, USA) and the WRS2 package (Mair, Wilcox and Patil 2024 version 1.1-6) in the R 4.3.1 environment.

## 4. Results

### 4.1. Characteristics

The characteristics of the study group, divided into LS ≤ 10 years and >10 years, are shown in Table 1. 

All study participants did not differ in height among themselves, but statistically significant differences were observed in BMI, WC, WHR, and SBP; these values were higher in the LS > 10 years group compared to the LS ≤ 10 years group. No differences were observed in DBP and PULSE.

### 4.2. Analysis of Covariance (ANCOVA)

To evaluate the association of NRF9.3 and LS on serum biochemical values, ANCOVA models were created, the results of which are shown in Table 2.

Diet quality had a statistically significant association on the value of parameters shown in Table 2 (excluding TSH), as expressed by the presence of significant differences in the mean values of GLU, FIL, TG/HDL, LDL/HDL, TC/HDL, non-HDL/HDL between groups of seniors for each diet quality value. In the TSH model (regardless of diet quality, the differences were statistically insignificant), and all other models, the FIL, TG/HDL, LDL/HDL, TC/HDL, and non-HDL/HDL shared a common pattern, with statistically significant differences for all diet quality values (excluding the smallest value in each of the models).

There was a strong upward trend in the GLU model; with an increase in the quality of the diet, the deltas between the seniority groups widened. No trends were observed in the other models (chaotic deltas).

### 4.3. Ordinal Multinomial Logistic Regression

The correlation of the quality of the firefighters’ diet, as expressed by the NRF9.3 index, was statistically significant for all lipid profile fraction proportions except TG/HDL, which was at the limit of significance. A negative correlation was observed between NRF9.3 and lipid profile fractional ratios; as the NRF9.3 increased, TG/HDL, LDL/HDL, TC/HDL, and non-HDL/HDL decreased.

The associations of different subsets with advanced glycation endproducts were also evaluated in the study, in different logistic regression models; the results are shown in Table 3. 

The quality of the diet, as expressed by the NRF9.3 index, is a key factor that alters the values of advanced glycation endproducts (AGEs) as one of the predictors of disordered glucose metabolism.

The AGE value was statistically significantly dependent on the quality of the diet, as expressed by the NRF9.3 index in each model, and on the TG/HDL ratio (*p* = 0.028, AIC = 191.627), but not dependent on LS in each model or on the other lipid profile ratios, LDL/HDL (*p* = 0.674, AIC = 202.352), non-HDL/HDL (*p* = 0.698, AIC = 202.382) and TC/HDL (*p* = 0.702, AIC = 202.386), respectively.

### 4.4. Lipid Ratios, Advanced Glycation Endproducts (AGE)

All differences in lipid profile components (i.e., TC, HDL, non-HDL, LDL, TG) between the LS ≤ 10 years and >10 years groups were statistically significant (Table 4). Each median concentration of individual lipid fractions was higher in the >10 years of seniority group. The exception was HDL cholesterol, the median of which was higher in the group with shorter LS, which is desirable for this cholesterol fraction. The values of the other parameters were statistically non-significantly different.

In this study, AGE values and lipid ratios were compared between the LS ≤ 10 years and LS > 10 years groups. The results of the Chi^2^ test are shown in Supplementary Table S6. In the analysis, we also tested a stricter TG/HDL ratio, which, according to Miller M. et al. [21], should be a maximum of 2.5 in men.

The study additionally examined the association of LS on the proportions of each lipid profile fraction. The results of the analysis are shown in Table 5. 

All the proportions of the lipid profile components were statistically significantly different between the seniority groups.

A comparison of the percentage of participants in each group LS ≤ 10 years and LS > 10 years on AGEs measured with the AGE reader and those whose cholesterol fraction indices were abnormal was conducted and calculated using Pearson’s chi-squared test of independence. The results are. as follows, respectively: (Pearson’s chi-squared independence test: *χ*^2^ = 5.67, *df* = 2, *p* = 0.059).

## 5. Discussion

The aim of our study was to evaluate the association of diet quality and seniority with biochemical indices, predictors of cardiometabolic diseases, among firefighters working rotating shifts. Night work, stress, and trauma occurring in this occupational group are factors that disrupt the metabolism of anabolic hormones such as insulin, and catabolic hormones such as cortisol, and also affect glucose levels, lipid profiles and blood pressure, the major CVD risk factors.

In our study, we observed a significant increase in systolic blood pressure (SBP) and pulse rate in the group of firefighters with longer service (LS > 10 years) compared to the group with shorter service (LS ≤ 10 years) (Table 1).

We demonstrated that diet quality has a significant association with GLU, FIL, and lipid profile component values, but not with TSH levels, between seniority groups for each diet quality value (excluding the single smallest value in the models) (Table 2). Statistically significant differences for all diet quality values (excluding the single smallest value in the models) were observed in the FIL, TG/HDL, LDL/HDL, TC/HDL, and non-HDL/HDL models. In the TSH model, regardless of diet quality, the differences were statistically insignificant. However, a strong upward trend was only observed in the GLU model; as the differences in diet quality increased, the deltas between the seniority groups widened. The other models lacked a similar trend as the deltas were chaotic.

The quality of the firefighters’ diet, as assessed by the NRF9.3 Index, and its relationship to anthropometric parameters, were described in a previously published article. Diet quality was not related to seniority or anthropometric parameters. In contrast, individual anthropometric parameters (body mass index, waist circumference, waist–hip ratio, body fat percentage, body fat mass) were significantly higher in the group of firefighters LS > 10 years compared to LS ≤ 10 years. Meta-analysis authors Souza R.V. et al. [22] noted that in addition to diet quality, changes in meal regularity and skipping breakfast were among the independent factors in weight gain and obesity, even despite adequate dietary energy supply among the participants. Considering the type of work done by professional firefighters, and the different times of departures for rescue and firefighting actions, the aforementioned risk factor also applies to the participants of our study.

Obesity and overweight affect the lipid profile as predictors of cardiometabolic diseases. There was a significant correlation between the quality of the firefighters’ diet and the ratios of the various cholesterol fractions (LDL/HDL, TC/HDL, and non-HDL/HDL), but not TG/HDL, which was at the significance borderline (Supplementary Table S3). A negative correlation was observed between NRF9.3 and the lipid profile fraction ratios; as NRF9.3 increased, TG/HDL, LDL/HDL, TC/HDL, and non-HDL/HDL ratios decreased. An individual ratio analysis of a patient’s lipid profile fractions may be the most accurate method used to assess the risk of cardiovascular atherosclerosis [23], MetS [24,25,26,27] and type II diabetes mellitus (T2DM) [28]. TC/HDL [29] or non-HDL/HDL [28,30,31] ratio analyses are considered the best single predictors of the risk of ischemic heart disease and, in the case of non-HDL/HDL [32,33,34], also of cardiovascular atherosclerosis. This may also contribute to earlier detection of abnormalities in a patient’s lipid profile, since even with normal LDL cholesterol levels, a patient may still be at increased risk for ischemic heart disease if the TC/HDL ratio is elevated [29]. This is related to the atherogenic and antiatherogenic properties of non-HDL cholesterol particles (which is the arithmetic sum of VLDL and LDL [35]) and HDL cholesterol particles, respectively; thus, the ratio has been recognized as a diagnostic tool for many diseases associated with dyslipidemia such as diabetes [36,37,38], MetS [32], carotid atherosclerosis [33,34], and also for the presence of abdominal aortic aneurysm [39].

The diets of firefighters in both seniority groups were characterized by > 10% dietary saturated fatty acids (13.77 (10.77, 15.83) for LS ≤ 10 years and 14.52 (12.50, 16.94) for LS > 10 years) [19], potentially accounting for the abnormal TC/HDL and LDL/HDL ratio in some study participants. The dishes prepared by the men, especially during the service, were high in animal fats such as processed meat, butter, full-fat cream, and cheese.

The reported lipid profile disturbances, and more specifically, the ratio of the different fractions, may also be due to an inadequate supply of dietary fiber, the proportion of which was <25 g/day (21.69 (17.40, 29.72) g for LS ≤ 10 years and 21.75 (16.45, 26.89) for LS > 10 years) in the diets of both seniority groups [19]. The reasons for men’s failure to consume their requirements for this ingredient are the predominance of highly refined grain products, i.e., wheat bread, white rice, breadcrumbs, and the low proportion of whole meal groats in the diet.

Proper lipid profile values among firefighters are especially important, as firefighters with elevated serum TC, LDL, TG and GLU levels did not achieve the minimum cardiorespiratory fitness requirements for firefighters [40]. Serum LDL cholesterol and TG levels are strongly related to physical fitness [41,42] and aerobic capacity [43]. Improving the quality of the diet will help to prevent the development of atherosclerosis, CVD and diseases that are components of the MetS, and as a result, maintain the aerobic capacity of the officers at an acceptable level.

Using multinomial ordinal logistic regression model analysis, we have shown that the quality of the firefighters’ diet (as expressed by the NRF9.3 index) is a significant factor in changing AGE values, one of the predictors of impaired glucose metabolism. In this model, AGE was significantly associated with TG/HDL, but not with other lipid profile indices or with the length of service (Table 3).

Advanced glycation endproducts (AGEs) are glucose-dependent products formed mainly as a result of glycemic, dicarbonyl, and oxidative stress. AGE concentrations can be elevated in disease states and accumulate in tissues, especially in association with proteins [44]. Advanced glycation endproducts co-occur in acute and chronic diseases, including diabetes [45,46,47], CVD [48,49], chronic kidney disease [50] and autoimmune diseases [51,52]. AGE measurements can provide information about pre-existing damage from chronic disease. We recorded elevated AGEs in only 18.03% and 14.49% of men in the LS ≤ 10 and >10 years groups, respectively, and the differences between the groups were not statistically significant (Supplementary Table S6).

The TG/HDL ratio counts as an alternative indicator of insulin resistance, a major cause of the development of T2DM; thus, the ratio can be a good predictor of its risk and is as reliable as the triglyceride/glucose ratio [53]. The statistical significance of AGEs with NRF9.3 that we obtained indicates the high importance of men’s diets to their health status and thus the safety of the Wroclaw population.

In turn, length of service was significantly associated with the components of the lipid profile, i.e., TC, HDL, non-HDL, LDL, and TG concentrations, but not with TSH, FIL, and GLU levels (Table 4). Each lipid fraction median concentration (Table 4) and the ratios between them (Table 5) were higher in the LS group > 10 years. The exception was HDL cholesterol, where the median was higher in the shorter tenure group, which is desirable as it has a strong and inverse association with CVD risk [28]. In addition, statistically significantly more men in the LS > 10 years group, had abnormal values of TG/HDL (Miller M.), TC/HDL, and LDL/HDL ratios, compared to men with shorter LS (Supplementary Table S6).

The results we collected are consistent with those published by Moffatt et al. [54]. These data highlight the need to monitor the health and well-being of professional firefighters throughout their employment. Metabolic and cardiovascular health deteriorates with increasing seniority, and thus with age; however, job duties remain the same throughout the entire period of service.

Taking into account the statistically significant differences in serum biochemical results between the seniority groups, it may be crucial to intervene with lifestyle education early in their careers to help slow the onset of lipid profile imbalances, thereby reducing the risk of CVD and T2DM, as further confirmed by the blood pressure data we collected from the subjects.

We did not observe differences in TSH, FIL and GLU levels between the seniority groups, but in the case of FIL and GLU, levels oscillated around the upper reference values in both seniority groups.

Based on our study results and those of other authors cited in this paper, special health care and specialized, systematic examinations should be extended, especially to firefighters with LS > 10 years. Even a minor slowdown in the rise in blood pressure due to the increasing age of men can reduce the risk, for example, of CVD associated with elevated serum blood pressure.

## 6. Limitations of the Study

The survey we conducted has some limitations. They relate to estimating the actual weights of individual products and dishes, especially those eaten in restaurants or bought to take away. Due to the high energy values of the diet, the requirements for individual dietary micronutrients and macronutrients are easier to meet, meaning that the calculated NRF9.3 index may not fully reflect the quality of the diet adhered to by study participants. In addition, due to the recruitment of men into the National Fire Service, it is not possible to disconnect the association of seniority and age of the participants with the biochemical results of the blood serum samples. Despite instructing volunteers taking part in the study about the length of the interval between the last meal and the blood draw in the lab, we are not sure if all men observed it. At the time of the anthropometric tests, the firefighters were on duty and making random calls to rescue and firefighting operations. When measuring blood pressure, some of the men may have had the so-called “white coat effect”; therefore, the values obtained may have been artificially inflated.

## 7. Conclusions

Firefighters’ diets, as assessed by the NRF9.3 index, had a significant association with predictors of insulin resistance and diabetes, i.e., GLU, FIL, TG/HDL ratio and AGE, as well as cardiometabolic predictors, i.e., to the atherogenic lipid (LDL, TC, non-HDL) and non-atherogenic lipid (HDL) ratios between seniority groups.

Only in the GLU model was a strong trend present; as the quality of the diet increased, the deltas between seniority groups increased. In the other models, the deltas were chaotic. The cardiometabolic predictors studied were significantly higher in firefighters with longer vs. shorter lengths of service.

Based on our findings and those of other authors cited in the paper, special health care and specialized, systematic examinations should be extended, especially to firefighters with longer service (e.g., >10 years). Even a slight slowing of the rise in blood pressure due to the increasing age of men can reduce the risk of, among other things, CVD associated with elevated serum blood pressure.

## Figures and Tables

**Table 1:** Comparison of anthropometric parameters of firefighters with shorter and longer lengths of service.

Parameters	$n,\;\bar{X}$ ± SD/Me (Q1–Q3)		U	z/w/t	*df*	r_/d_	*p* ^z/w/t^
	LS ≤ 10 Years	LS > 10 Years					
Age [years]	62, 29.00 (26.00–33.00)	71, 40.49 (41.00–37.00)	268.50	−8.73 ^z^	-	**0.12 ^r^**	**<0.001 ^z^**
H [cm]	62, 180.6 ± 5.4	71, 179.2 ± 6.8	-	−1.33 ^w^	131	0.23 ^d^	0.187 ^w^
BMI [kg/m^2^]	62, 25.40 (23.80–26.80)	71, 28.10 (25.90–30.00)	1091.50	−5.00 ^z^	-	**0.50 ^r^**	**<0.001 ^z^**
WC [cm]	62, 89.00 (82.00–93.00)	71, 96.50 (89.00–103.00)	1175.00	−4.23 ^z^	-	**0.53 ^r^**	**<0.001 ^z^**
WHR	62, 0.84 (0.81–0.86)	71, 0.92 (0.89–0.96)	442.50	−7.93 ^z^	-	**0.20 ^r^**	**<0.001 ^z^**
DBP [mm/Hg]	62, 79.00 (73.00–89.00)	69, 83.00 (77.00–89.00)	1775.00	−1.68 ^z^	-	0.55 ^r^	0.094 ^z^
SBP [mm/Hg]	62, 134.4 ± 14.4	69, 139.5 ± 14.3	-	−2.03 ^t^	129	0.36 ^d^	0.044 ^t^
PULSE [bpm]	61, 71.3 ± 12.8	69, 71.2 ± 11.5	-	0.02 ^t^	128	0.10 ^d^	0.983 ^t^

*n*—observations number, $\bar{X}$ ± SD/Me (Q1–Q3)—mean ± standard deviation/median (quartile 1–quartile 3), U—the value of the M–W test statistic to r_g_, *z*—the test value of the M–W test, ***w***—value of the *t*-student test statistic with Welch correction, *t*—value of the *t*-student test statistic for independent groups), *df*—degrees of freedom, r—r_g_—a measure of the magnitude of the Wald effect (non-directional rank-dual correlation formula; *r_g_*: 0.01–0.20 -> little effect, 0.21–0.50 -> medium effect, > 0.50 -> large effect), for the M–W test, d—dCohen—Cohen’s d effect size measure of the *t*-student test (with and without Welch correction; dCohen: 0.01–0.20 -> little effect, 0.21–0.50 -> medium effect, >0.50 -> large effect), *p^z^*—statistical significance level of the M–W test, *p^w^*—level of statistical significance of the Welch corrected Student’s *t*-test, *p^t^*—statistical significance level of the *t*-student test, assumed α = 0.05; H—height, BMI—body mass Index, WC—waist circumference, WHR—waist–hip ratio, DBP—diastolic blood pressure, SBP—systolic blood pressure.

**Table 2:** Comparison of the association of the difference in diet quality on parameter values expressed as averages between short and long lengths of service according to non-parametric ANCOVA.

NRF9.3	LS ≤ 10 Years	LS > 10 Years	Δ	SE	95% CI−	95% CI+	A	*p* ^A^
Model: 1. GLU ← LS (≤10 years, >10 years) + Age (≤35 years, >35 years) + BMI (18.5–24.9, >24.9, <18.5) + + WHR (<1.0 -> 0, ≥1.0 -> 1) + NRF9.3								
535.087	14	12	0.650	3.113	−9.088	10.388	0.196	0.848
631.149	32	32	2.950	1.353	−0.705	6.605	2.180	**0.036**
680.909	37	34	4.006	1.177	0.833	7.179	3.404	**0.002**
712.631	34	36	4.409	1.413	0.590	8.228	3.121	**0.004**
801.642	13	21	5.530	1.904	−0.061	11.121	2.904	**0.012**
Model: 2. FIL ← LS (≤10 years, >10 years) + Age (≤35 years, >35 years) + BMI (18.5–24.9, >24.9, <18.5) + + WHR (<1.0 -> 0, ≥1.0 -> 1) + NRF9.3								
535.087	14	12	0.650	3.323	−9.088	10.388	0.196	0.848
631.149	32.	32	2.950	1.353	−0.705	6.605	2.180	**0.036**
680.909	37	34	4.006	1.177	0.833	7.178	3.404	**0.002**
712.631	34.36	4.409	1.413	1.413	0.590	8.228	3.121	**0.004**
801.642	13	21	5.530	1.904	−0.061	11.121	2.904	**0.012**
Model: 3. TSH ← LS (≤10 years, >10 years) + Age (≤35 years, >35 years) + BMI (18.5–24.9, >24.9, <18.5) + + WHR (<1.0 -> 0, ≥ 1.0 -> 1) + NRF9.3								
535.087	15	12	−0.209	0.295	−1.114	0.695	0.711	0.493
631.149	32	26	−0.252	0.201	−0.807	0.302	1.256	0.221
680.651	37	29	−0.235	0.189	−0.708	0.239	1.369	0.183
712.631	36	30	−0.202	0.189	−0.730	0.323	1.070	0.296
801.642	13	17	−0.098	0.234	−0.765	0.570	0.418	0.681
Model: 4. TG/HDL ← LS (≤10 years, >10 years) + Age (≤35 years, >35 years) + BMI (18.5–24.9, >24.9, <18.5) + + WHR (<1.0 -> 0, ≥1.0 -> 1) + NRF9.3								
535.087	14	12	0.701	0.620	−1.149	2.550	1.130	0.280
631.149	32	32	1.531	0.272	0.795	2.268	5.628	**<0.001**
680.909	37	34	1.274	0.525	2.024	4.654	4.654	**<0.001**
712.631	34	36	1.119	0.260	0.408	1.830	4.302	**<0.001**
801.642	13	21	0.761	0.287	−0.097	1.619	2.649	**0.021**
Model: 5. LDL/HDL ← LS (≤10 years, >10 years) + Age (≤35 years, >35 years) + BMI (18.5–24.9, >24.9, <18.5) + + WHR (<1.0 -> 0, ≥1.0 -> 1) + NRF9.3								
535.087	14	12	0.496	0.467	−0.863	1.855	1.062	0.305
631.149	32	32	0.752	0.217	0.165	1.340	3.459	**0.001**
680.909	37	34	0.736	0.172	0.274	1.199	4.290	**<0.001**
712.631	34	36	0.903	0.174	0.432	1.373	5.185	**<0.001**
801.642	13	21	1.041	0.352	−0.038	2.119	2.955	**0.013**
Model: 6. TC/HDL ← LS (≤10 years, >10 years) + Age (≤35 years, >35 years) + BMI (18.5–24.9, >24.9, <18.5) + + WHR (<1.0 -> 0, ≥1.0 -> 1) + NRF9.3								
535.087	14	12	0.766	0.569	−0.888	2.420	1.347	0.198
631.149	32	32	1.094	0.281	0.336	1.853	3.892	**<0.001**
680.909	37	34	1.054	0.217	0.467	1.641	4.867	**<0.001**
712.631	34	36	1.179	0.216	0.589	1.768	5.453	**<0.001**
801.642	13	21	1.272	0.390	0.071	2.474	3.263	**0.008**
Model: 7. non-HDL/HDL ← LS (≤ 10 years, >10 years) + Age (≤35 years, >35 years) + BMI (18.5–24.9, >24.9, <18.5) + + WHR (<1.0 -> 0, ≥1.0 -> 1) + NRF9.3								
535.087	14	12	0.766	0.569	−0.888	2.420	1.367	0.198
631.149	32	32	1.070	0.281	0.312	1.829	3.808	**<0.001**
680.909	37	34	1.032	0.218	0.443	1.622	4.741	**<0.001**
712.631	34	36	1.157	0.220	0.559	1.755	5.264	**<0.001**
801.642	13	21	1.272	0.390	0.071	2.474	33.263	**0.008**

Δ—difference (difference in averages between short and long tenure), SE—standard error, 95% CI—lower value of the 95% confidence interval, 95% CI+—upper value of 95% confidence interval, A—the value of the test statistic for the nonparametric ANCOVA, *p*^A^—the statistical significance of the test for the nonparametric ANCOVA, NRF9.3—Nutrient Rich Food Index 9.3, LS—length of service (≤10 years -> 0, >10 years -> 1), Age (≤35 years -> 0, >35 years -> 1), BMI (18.5–24.9 -> 0, >24.9 -> 1, <18.5 -> 2), WHR (<1.0 -> 0, ≥1.0 -> 1). GLU—glucose level, FIL—fasting insulin levels, TSH—thyrotropin hormone levels, TG/HDL—triglyceride and HDL cholesterol ratio, LDL/HDL—LDL and HDL cholesterol ratio, TC/HDL—total and HDL cholesterol ratio, non-HDL/HDL—non-HDL and HDL cholesterol ratio.

**Table 3:** Comparison of the significance of parameters (NRF9.3, LS, and the proportion of each lipid profile fraction) on the advanced glycation endproducts tested with an AGE reader device.

Effect	Level Effect	Column	Evaluation	SE	W	95% CI−	95% CI+	*p*
Model: 1. AGE ← LS (≤10 years, >10 years) + NRF9.3 + TG/HDL								
absolute term 1	-	1	1.855	1.600	1.344	−0.282	4.992	0.246
absolute term 2	-	2	4.667	1.669	7.819	1.396	7.938	0.005
NRF9.3	-	3	−0.005	0.002	4.634	−0.009	−<0.001	**0.031**
TG/HDL	-	4	0 237	0.217	1.190	−0.188	0.662	**0.028**
LS (≤10 years, >10 years)	0	5	−0.094	0.222	0.178	−0.523	0.341	0.673
scale	-	-	1.000	<0.001	-	1.000	1.000	-
Model: 2. AGE ← LS (≤ 10 years, >10 years) + NRF9.3 + LDL/HDL								
absolute term 1	-	1	2.341	1.644	2.028	−0.881	5.562	0.154
absolute term 2	-	2	5.125	1.715	8.935	1.764	8.485	0.003
NRF9.3	-	3	−0.005	0.002	4.329	−0.009	−<0.001	**0.037**
LDL/HDL	-	4	−0.096	0.227	0.177	−0.541	0.350	0.674
LS (≤10 years, >10 years)	0	5	−0.281	0.211	1.776	−0.694	0.132	0.183
scale	-	-	1.000	<0.001	-	1.000	1.000	-
Model 3. AGE ← LS (≤10 years, >10 years) + NRF9.3 + TC/HDL								
absolute term 1	-	1	2.393	1.753	1.863	−1.043	5.829	1.172
absolute term 2	-	2	5.177	1.820	8.090	1.610	8.743	0.004
NRF9.3	-	3	−0.005	0.002	4.302	−0.009	−<0.001	**0.038**
TC/HDL	-	4	−0.073	0.190	0.146	−0.445	0.300	0.702
LS (≤10 years, >10 years)	0	5	−0.281	0.213	1.747	−0.698	0.136	0.186
scale	-	-	1.000	0.001	-	1.000	1.000	-
Model 4. AGE ← LS (≤10 years, >10 years) + NRF9.3 + non-HDL/HDL								
absolute term 1	-	1	2.326	1.659	1.966	−0.926	5.578	0.161
absolute term 2	-	2	5.110	1.728	8.739	1.722	8.497	0.003
NRF9.3	-	3	−0.005	0.002	4.307	−0.009	−<0.001	**0.038**
non-HDL/HDL	-	4	−0.074	0.190	0.151	−0.445	0.298	0.698
LS (≤10 years, >10 years)	0	5	−0.281	0.213	1.753	−0.698	0.135	0.186
scale	-	-	1.000	<0.001	-	1.000	1.000	-

SE—standard error, W—Wald test statistic, 95% CI−—lower value of the 95% confidence interval Cl, 95% CI+—upper value of 95% confidence interval, *p*—level of statistical significance of the Wald test, assumed α = 0.05, AGE—advanced glycation endproducts tested with an AGE reader device, NRF9.3—Nutrient Rich Food Index 9.3, LS—length of service, TG/HDL—triglyceride and HDL cholesterol ratio, LDL/HDL—LDL and HDL cholesterol ratio, TC/HDL—total and HDL cholesterol ratio, non-HDL/HDL—non-HDL and HDL cholesterol ratio.

**Table 4:** Comparison of concentrations of lipid profile, TSH, fasting insulin, and fasting glucose between LS ≤ 10 years and LS > 10 years groups.

Parameters	$n,\;\bar{X}$ ± SD/Me (Q1–Q3)		U	z	r_g_	*p*-ad^z^
	LS ≤ 10 Years	LS > 10 Years				
TC [mg/dL]	50, 184.00 (166.00–194.00)	58, 211.50 (184.00–254.00)	795.50	0.06	**0.55**	**0.003**
HDL [mg/dL]	50, 58.00 (51.70–66.10)	58, 51.30 (45.60–56.70)	956.50	3.04	**0.66**	**0.033**
non-HDL [mg/dL]	50, 122.00 (106.00–140.00)	58, 151.70 (132.00–196.70)	699.50	−4.62	**0.48**	**0.004**
LDL [mg/dL]	50, 105.18 (90.24–119.00)	58, 123.75 (108.20–167.90)	805.00	−3.97	**0.56**	**0.005**
TG [mg/dL]	50, 78.00 (67.00–103.00)	58, 118.50 (96.00–158.00)	689.00	−4.68	**0.48**	**0.006**
TSH [μIU/mL]	41, 1.35 (0.95–2.04)	54, 1.12 (0.85–1.45)	962.00	1.09	0.87	1.000
FIL [μIU/mL]	41, 6.80 (5.27–8.72)	54, 9.62 (6.06–13.35)	781.50	−2.40	0.71	0.191
GLU [mg/dL]	50, 87.00 (84.00–89.00)	58, 90.00 (87.00–96.00)	987.00	−2.86	0.68	0.063

*n*—observations number, $\bar{X}$ ± SD/Me (Q1–Q3)—mean ± standard deviation/median (quartile 1—quartile 3), U—the value of the M–W test statistic to r_g_, z—value of the M–W test statistic, r—r_g_—a measure of the magnitude of the Wald effect (non-directional rank-dual correlation formula; *r_g_*: 0.01–0.20 -> little effect, 0.21–0.50 -> medium effect, >0.50 -> large effect) for the M–W test, *p*-ad^z^—adjusted statistical significance level (Dunn–Sidak–Holm correction was imposed on level of statistical significance), assumed α = 0.05, TC—total cholesterol levels, HDL—high-density lipoprotein cholesterol levels, non-HDL—non-HDL cholesterol levels, LDL—low-density lipoprotein cholesterol levels, TG—triglyceride levels, TSH—thyrotropin hormone levels, FIL—fasting insulin levels, GLU—glucose.

**Table 5:** Comparison of the ratio of concentrations of individual lipid profile fractions between LS ≤ 10 years and LS > 10 years groups.

Lipid Ratio	Me (Q1–Q3)			z	r_g_	*p*-ad^z^
	LS ≤ 10 Years (*n* = 50)	LS > 10 Years (*n* = 58)	U			
TG/HDL	1.38 (0.97–1.85)	2.34 (1.69–3.40)	749.00	−4.31	**0.52**	**0.007**
LDL/HDL	1.80 (1.41–2.25)	2.63 (3.39–4.93)	772.00	−4.17	**0.53**	**0.008**
TC/HDL	3.04 (2.71–3.55)	4.18 (3.39–4.93)	726.00	−4.46	**0.50**	**0.009**
non-HDL/HDL	2.07 (1.71–2.61)	3.18 (2.39–3.93)	734.00	−4.41	**0.51**	**0.010**

Me (Q1–Q3)—median (quartile 1—quartile 3), U—the value of the M–W test statistic to r_g_, z—value of the M–W test statistic, r_g_ = a measure of the magnitude of the Wald effect (non-directional rank-dual correlation formula; r_g_: 0.01–0.20 -> little effect, 0.21–0.50 -> medium effect, >0.50 -> large effect) for the M–W test, *p*-ad^z^—level of statistical significance (a Bonferroni correction was applied to multiple comparisons), assumed α = 0.05; TG/HDL—triglyceride and HDL cholesterol ratio, LDL/HDL—LDL and HDL cholesterol ratio, TC/HDL—total and HDL cholesterol ratio, non-HDL/HDL—non HDL and HDL cholesterol ratio.

## Data Availability

The original contributions presented in the study are included in the article/Supplementary Material, further inquiries can be directed to the corresponding author.

## Supplementary Materials

The following supporting information can be downloaded at: https://www.mdpi.com/article/10.3390/nu16152467/s1, Figures S1–S5: Checking the assumption of no outliers (using box-and-whisker plots, all values less than Q1 + 1.5 × IQR and greater than that up to Q3 + 1.5 × IQR) for logistic regression for the Results 4.3. Ordinal multinomial logistic regression. NRF9.3 and cholesterol fraction ratio.; Figures S6–S13: Testing the assumption of no collinearity of predictors (Spearman’s correlation) for logistic regression for the Results 4.3. Multinomial ordinal logistic regression. NRF9.3 versus cholesterol fraction ratio; Table S1: Checking the assumption of normality of distribution (for n ≤ 50 using the Shapiro-Wilk test and for n > 50 using the Kolomogrov-Smirnov test with Lilliefors correction) for the Results 4.1 Characteristics and the Results 4.4. (Table 1) Lipid ratio, AGE (Table 4, Table 5); Table S2: Checking the assumption of normality of distribution (using Kolomogrov-Smirnov test with Lilliefors correction) for the Results 4.2. ANCOVA (Table 2); Table S3: Testing the assumption of non-collinearity of predictors (using Spearman’s correlation) for logistic regression for the Results 4.3. Multinomial ordinal logistic regression (MOLR) (Table 3). NRF9.3 versus cholesterol fraction ratio; Table S4: Testing the assumption of logit between predictors and the logit of the predictors (using the Box-Tidwell test) for logistic regression fot the Results 4.3. Multinomial ordinal logistic regression. Effects of LS, NRF9.3, cholesterol fraction ratio on AGEs; Table S5: Comparison of subsets of variables (NRF9.3, LS and lipid fraction levels) with AGEs in different regression models according to Wald test for logistic regression fot the Results 4.3. Multinomial ordinal logistic regression (MOLR). Effects of LS, NRF9.3, cholesterol fraction ratio on AGEs.; Table S6: Comparison of the percentage of participants in each of the LS ≤ 10 years and LS > 10 years group on AGEs measured with AGE Reader and whose cholesterol fraction ratios were abnormal calculated by Pearson’s Chi2 correlation.
