# Supplementary material for: Association of the Length of Service in the 24/48 Shift of Firefighters of the State Fire Service in Wroclaw on Selected Serum Biochemical Parameters of Nutritional Status
Source: Nutrients. 2024 Jul 29;16(15):2467. doi: 10.3390/nu16152467 (PMC11314308; doi:10.3390/nu16152467)
Supplement: Supplementary file 1 [file nutrients-16-02467-s001.zip › nutrients-3067803-supplementary.pdf]

SUPLEMENT

Supplementary Table S1 Checking the assumption of normality of distribution (for n ≤ 50 using the Shapiro-Wilk test and for n > 50 using the Kolomogrov-Smirnov test with Lilliefors correction) for the Results 4.1 Characteristics and the Results 4.4. (Table 1) Lipid ratio, AGE (Table 4, Table 5).

				**LS ≤ 10 y**			**LS > 10 y**
**To the Results, Table 1**	**Variable**	**n**	***SW/D***	***p^{SW/D}***	**n**	***SW/D***	***p^{SW/D}***
4.1.Characteristics, T1	Age						
4.1.Characteristics, T1	*H	52	0.12^D	0.091^D	71	0.06^D	0.108^D
4.1.Characteristics, T1	BMI	62	0.06^D	0.897^D	71	0.14^D	$<0.001^D$
4.1.Characteristics, T1	WC.	62	0.07^D	0.228^D	71	0.09^D	0.004^D
4.1.Characteristics, T1	WHR	62	0.15^D	$<0.001^D$	71	0.14^D	0.094^D
4.1.Characteristics, T1	DBP	62	0.09^D	0.048^D	69	0.09^D	0.002^D
4.1.Characteristics, T1	*SBP	62	0.12^D	0.240^D	69	0.08^D	0.743^D
4.1.Characteristics, T1	*PULSE	61	0.09^D	0.120^D	69	0.10^D	0.382
4.4. Lipid ratio, AGE, T4	TC	50	0.90^{SW}	$<0.001^{SW}$	58	0.98^{SW}	0.434^{SW}
4.4. Lipid ratio, AGE, T4	HDL	50	0.99^{SW}	0.996^{SW}	58	0.86^{SW}	$<0.001^{SW}$
4.4. Lipid ratio, AGE, T4	non-HDL	50	0.90^{SW}	$<0.001^{SW}$	58	0.96^{SW}	0.077^{SW}
4.4. Lipid ratio, AGE, T4	LDL	50	0.92^{SW}	0.002^{SW}	58	0.96^{SW}	0.040^{SW}
4.4. Lipid ratio, AGE, T4	TG	50	0.79^{SW}	$<0.001^{SW}$	58	0.88^{SW}	$<0.001^{SW}$
4.4. Lipid ratio, AGE, T4	TSH	41	0.95^{SW}	0.070^{SW}	54	0.72^{SW}	$<0.001^{SW}$
4.4. Lipid ratio, AGE, T4	FIL	41	0.90^{SW}	0.002^{SW}	54	0.78^{SW}	$<0.001^{SW}$
4.4. Lipid ratio, AGE, T4	GLU	50	0.97^{SW}	0.305^{SW}	58	0.95^{SW}	0.014^{SW}
4.4. Lipid ratio, AGE, T5	TG/HDL	50	0.73^{SW}	$<0.001^{SW}$	58	0.83^{SW}	$<0.001^{SW}$
4.4. Lipid ratio, AGE, T5	LDL/HDL	50	0.90^{SW}	$<0.001^{SW}$	58	0.94^{SW}	0.006^{SW}
4.4. Lipid ratio, AGE, T5	TC/HDL	50	0.86^{SW}	$<0.001^{SW}$	58	0.97^{SW}	0.127^{SW}
4.4. Lipid ratio, AGE, T5	non-HDL/HDL	50	0.87^{SW}	$<0.001^{SW}$	58	0.97^{SW}	0.127^{SW}

T – table, n – number of ovservation, SW – Shapiro-Wilk test value, D - Kolomogrov-Smirnov test value with Lilliefors correction, p^{SW} – level of statistical significance of the Shapiro-Wilk test, p^D - Kołomogrov-Smirnov test with Lilloiefors correction, LS - length of service, y – year, * testing the assumption of homogeneity of variance (Leven test): H (F=2.1, df=131, p=0.004), SBP (F=0.01, df=129, p=0.933), PULSE (F=1.07, df=128, p=0.303), F – Leven's test value, df – degrees od freedom, p^F – level of statistical significance of the Leven's test, AGE – advanced glycation end products.

Supplementary Table S2 Checking the assumption of normality of distribution (using Kolomogrov-Smirnov test with Lilliefors correction) for the Results 4.2. ANCOVA (Table 2).

**To the Results, Table 2**	**Variable**	**n**	***D***	***p^D***
4.2. ANCOVA, T2	TG/HDL	108	0.16	<0.001
4.2. ANCOVA, T2	LDL/HDL	108	0.13	<0.001
4.2. ANCOVA, T2	TC/HDL	108	0.12	<0.001
4.2. ANCOVA, T2	non-HDL/HDL	108	0.11	<0.001

n – number of observation, ***D*** – Kolomogrov-Smirnov test with Lillieforse correction, p^D **–** Kołomogrov-Smirnov test with Lilloiefors correction

Supplementary Figures S1–S5 Checking the assumption of no outliers (using box-and-whisker plots, all values less than Q1+1.5 x IQR and greater than that up to Q3 + 1.5 x IQR) for logistic regression for the Results 4.3. Ordinal multinomial logistic regression. NRF9.3 and cholesterol fraction ratio.

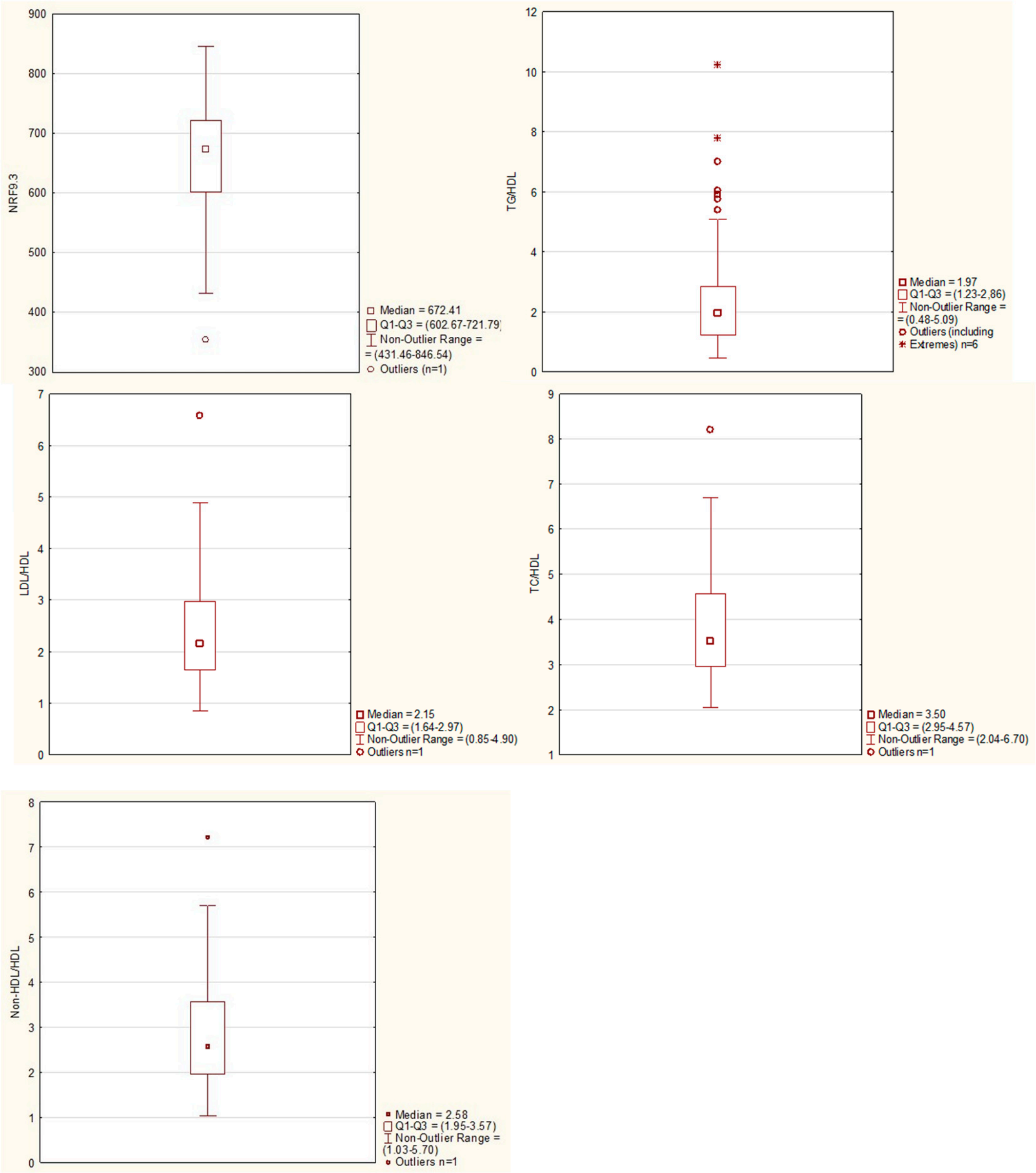

Supplementary Table S3. Testing the assumption of non-collinearity of predictors (using Spearman's correlation) for logistic regression for the Results 4.3. Multinomial ordinal logistic regression (MOLR) (Table 3). NRF9.3 versus cholesterol fraction ratio.

To the Results, Table	Model	Predictors	*n*	*R*	*T(N-2)*	*p*
4.3. MOLR, T3	1.	NRF9.3 & TG/HDL	100	- 0,19	- 1.95	0.054*
4.3. MOLR, T3	2.	NRF9.3 & LDL/HDL	106	- 0.20	- 2.04	**0.044**
4.3. MOLR, T3	3.	NRF9.3 & TC/HDL	106	- 0.23	- 2.40	**0.018**
4.3. MOLR, T3	4.	NRF9.3 & non-HDL/HDL	106	- 0.23	- 2.43	**0.017**

n – observation of number, *R* - Spearman correlation coefficient, *T(N-2)* - test statistic value, *p* - Spearman's correlation statistical significance level (*0.054 –marginal level of statistical significance), assumed α = 0.05. NRF9.3 – Nutrient Rich Food Index 9.3, TG/HDL – triglyceride and HDL cholesterol ratio, LDL/HDL – LDL and HDL cholesterol ratio, TC/HDL – total and HDL cholesterol ratio, non-HDL/HDL – non HDL and HDL cholesterol ratio.

Supplementary Figures S6–S13. Testing the assumption of no collinearity of predictors (Spearman's correlation) for logistic regression for the Results 4.3. Multinomial ordinal logistic regression. NRF9.3 versus cholesterol fraction ratio.

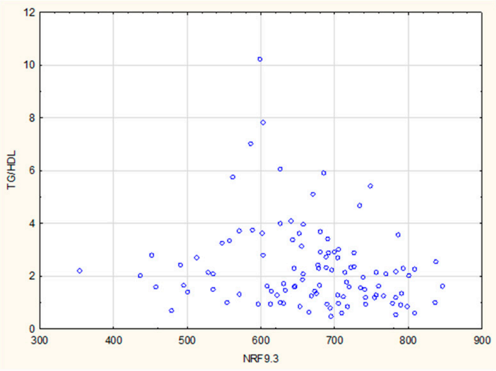

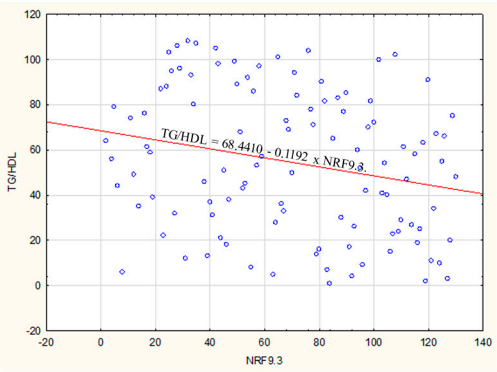

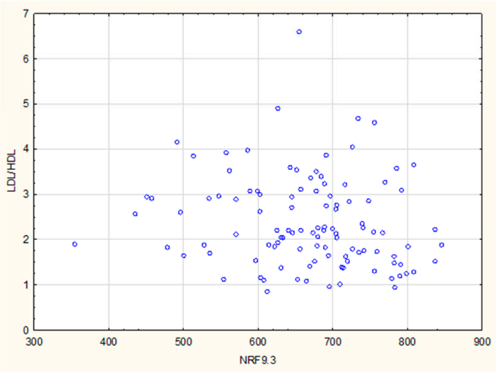

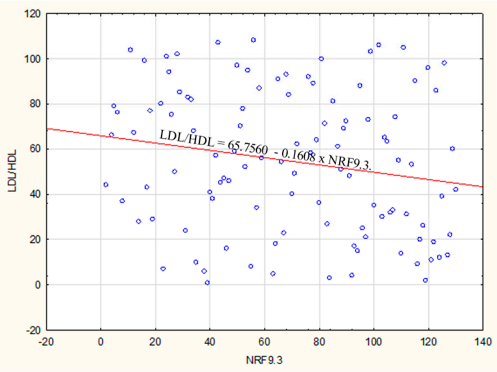

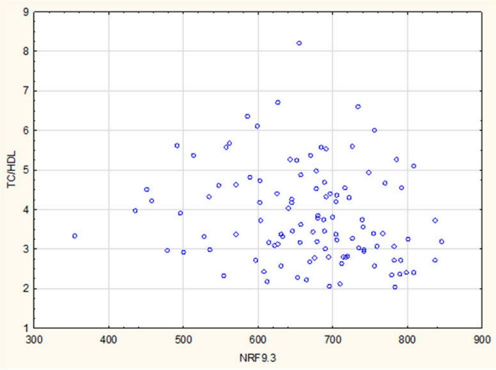

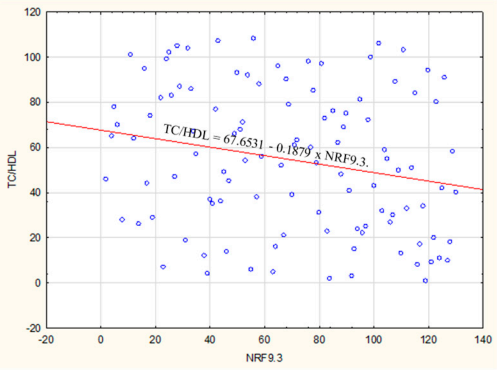

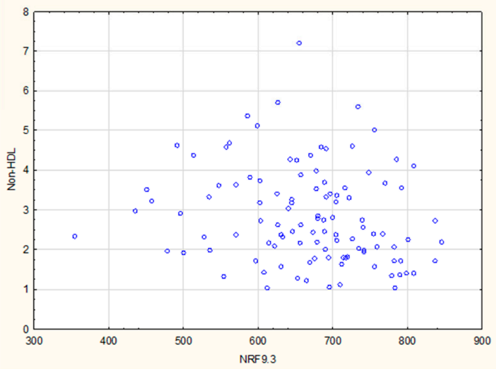

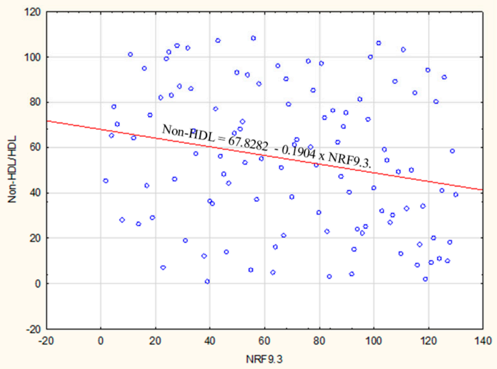

NRF9.3 – Nutrient Rich Food Index 9.3, TG/HDL – triglyceride and HDL cholesterol ratio, LDL/HDL – LDL and HDL cholesterol ratio, TC/HDL – total and HDL cholesterol ratio, non-HDL/HDL – non HDL and HDL cholesterol ratio

Supplementary Table S4 Testing the assumption of logit between predictors and the logit of the predictors (using the Box-Tidwell test) for logistic regression fot the Results 4.3. Multinomial ordinal logistic regression. Effects of LS, NRF9.3, cholesterol fraction ratio on AGEs.

Effect	W	df	p^{B-T}
**Model: 1. AGE ← LS (≤ 10 years, >10 years) + NRF9.3 + TG/HDL**			
absolute term	68.62	2	**<0.001**
NRF9.3	0.18	1	0.670
NRF9.3*ln_NRF9.3	0.20	1	0.652
TG/HDL	0.43	1	0.514
TG/HDL*ln_TG/HDL	0.22	1	0.643
**Model: 2. AGE ← LS (≤ 10 years, >10 years) + NRF9.3 + LDL/HDL**			
absolute term	73.81	2	**<0.001**
NRF9.3	0.25	1	0.620
NRF9.3*ln_NRF9.3	0.74	1	0.601
LDL/HDL	0.04	1	0.833
LDL/HDL*ln_LDL/HDL	0.04	1	0.834
**Model 3. AGE ← LS (≤ 10 years, >10 years) + NRF9.3 + TC/HDL**			
absolute term	73.07	2	**<0.001**
NRF9.3	0.32	1	0.574
NRF9.3*ln_NRF9.3	0.35	1	0.556
TC/HDL	1.07	1	0.300
TC/HDL*ln_TC/HDL	1.07	1	0.301
**Model 4. AGE ← LS (≤ 10 years, >10 years) + NRF9.3 + non-HDL/HDL**			
absolute term	73.15	2	**<0.001**
NRF9.3	0.31	1	0.577
NRF9.3*ln_NRF9.3	0.34	1	0.559
non-HDL/HDL	0.96	1	0.327
non-HDL/HDL*ln_non-HDL/HDL	0.96	1	0.327

W – Wald test statistic, *df* - degree of freedom, p^{B-T} - statistical significance level of Box-Tidwell test, assumed α=0.05, AGE - cardiovascular disease risk tested by AGE Reader device, LS – length of service, NRF9.3 – Nutrient Rich Food Index 9.3, TG/HDL – triglyceride and HDL cholesterol ratio, LDL/HDL – LDL and HDL cholesterol ratio, TC/HDL – total and HDL cholesterol ratio, non-HDL/HDL – non HDL and HDL cholesterol ratio

Supplementary Table S5 Comparison of subsets of variables (NRF9.3, LS and lipid fraction levels) with AGEs in different regression models according to Wald test for logistic regression fot the Results 4.3. Multinomial ordinal logistic regression (MOLR). Effects of LS, NRF9.3, cholesterol fraction ratio on AGEs.

Effect	*W*	*df*	p^W
**Model: 1. AGE ← LS (≤ 10 years, >10 years) + NRF9.3 + TG/HDL**			
absolute term	69.51	2	>0.001
NRF9.3	4.63	1	0.031
TG/HDL	1.19	1	0.028
LS (≤ 10 years, >10 years)	0.18	1	0.673
**Model: 2. AGE ← LS (≤ 10 years, >10 years) + NRF9.3 + LDL/HDL**			
absolute term	74.11	2	<0.001
NRF9.3	4.33	1	0.037
LDL/HDL	0.18	1	0.674
LS (≤ 10 years, >10 years)	1.18	1	0.183
**Model 3. AGE ← LS (≤ 10 years, >10 years) + NRF9.3 + TC/HDL**			
absolute term	74.16	2	<0.001
NRF9.3	4.30	1	0.038
TC/HDL	0.15	1	0.702
LS (≤ 10 years, >10 years)	1.75	1	0.186
**Model 4. AGE ← LS (≤ 10 years, >10 years) + NRF9.3 + non-HDL/HDL**			
absolute term	74.16	2	<0.001
NRF9.3	4.31	1	0.038
non-HDL/HDL	0.15	1	0.698
LS (≤ 10 years, >10 years)	1.75	1	0.186

W - Wald test statistic, *df* - degree of freedom, p^W - statistical significance level of Wald test, assumed, AGE - Advanced Glycation Endproducts tested by AGE Reader device, LS – length of service, NRF9.3 – Nutrient Rich Food Index 9.3, TG/HDL – triglyceride and HDL cholesterol ratio, LDL/HDL – LDL and HDL cholesterol ratio, TC/HDL – total and HDL cholesterol ratio, non-HDL/HDL – non HDL and HDL cholesterol ratio.

Supplementary Table S6. Comparison of the percentage of participants in each of the LS ≤ 10 years and LS > 10 years group on AGEs measured with AGE Reader and whose cholesterol fraction ratios were abnormal calculated by Pearson 's Chi^2 correlation.

	n [%]				
	LS ≤ 10 years	***LS > 10 years***	***df***	***χ 2***	***p/p-ad***
AGE	***(n=61)***	***(n=69)***			
0	10 [16.39]	24 [34.78]			
1	40 [65.57]	35 [50.72]	2	5.67	0.059
2	11 [18.03]	10 [14.49]			
LIPID RATIO	***(n=50)***	***(n=58)***			
TG/HDL	4 [8.0]	11 [19.0]		2.70	0.045*
TG/HDL (Miller M.)	7 [14.0]	27 [46.0]		13.19	**0.010***
LDL/HDL	10 [20.0]	26 [44.8]	1	0.85	**0.020***
TC/HDL	6 [12.0]	20 [34.5]		7.43	**0.030***
non-HDL/HDL	8 [16.0]	22 [37.9]		6.64	0.032*

AGE - the value of glycation end products (0 - lower, 1 - moderate, 2 - increased), LS – length of service, n - the number of people in the group, y - the number of abnormal participants cholesterol fraction ratios, df - degrees of freedom, χ 2 – Chi2 consistency, p – statistical significance (statistically significant result for p<0.05), p-ad - p-adjusted (a Bonfferoni-Hochberg correction was applied to the "p" values; statistically significant result for p<0.032), TG/HDL – triglyceride and HDL cholesterol ratio, TG/HDL (Miller M.) – triglyceride and HDL cholesterol ratio according to Miller M. et al. [14]; LDL/HDL – LDL and HDL cholesterol ratio, TC/HDL – total and HDL cholesterol ratio, non-HDL/HDL – non HDL and HDL cholesterol ratio; * - p-.ad
